# CKAP4 silencing impairs growth and survival of esophageal adenocarcinoma cells in vitro

**DOI:** 10.1016/j.tranon.2026.103100

**Published:** 2026-11-04

**Authors:** Beate Limberger, Alexandra Ludwig, Philipp Walther, Chuang Yang, Andreas Teufel, Timo Itzel, Patrick S. Plum, Orestis Lyros, Stefan Niebisch, Ines Gockel, René Thieme

**Affiliations:** a Department of Visceral, Transplant, Thoracic and Vascular Surgery, University Hospital Leipzig, Germany; b Department of Hepatology, Department of Clinical Bioinformatics, University Hospital of Mannheim, Germany; c Department of Internal Medicine A: Gastroenterology, Endocrinology, Nephrology, Rheumatology, and Nutritional Medicine, University Hospital Greifswald, Germany; d Department of General, Visceral and Transplantation Surgery, University Hospital Tübingen, Tübingen, Germany; e 4th Department of Surgery, Attikon University Hospital, National and Kapodistrian University of Athens, Greece; f Department of Visceral Surgery, Clarunis, University Digestive Health Care Center Basel, St. Clara Hospital and University Hospital Basel, Basel, Switzerland

**Keywords:** Esophageal adenocarcinoma, Barrett´s esophagus, Cytoskeleton-associated protein 4, Dickkopf-protein 1

## Abstract

**Background:**

Esophageal adenocarcinoma (EAC) is the predominant histologic subtype of esophageal cancer in Western countries and has a poor prognosis. Cytoskeleton-associated protein 4 (CKAP4), a transmembrane receptor involved in proliferation, migration, and signaling, has been implicated in several cancers, but its role in EAC remains unclear. We investigated CKAP4 expression and cellular changes associated with CKAP4 depletion in EAC cell models.

**Methods:**

CKAP4 expression was characterized using an in vitro Barrett’s esophagus (BE) model of EAC progression. CKAP4 was silenced by siRNA in OE33 and OE19 EAC cells. Functional and molecular effects were assessed, and transcriptional changes were analyzed by RNA sequencing.

**Results:**

CKAP4 expression varied across Barrett’s neoplastic progression, with highest total expression in dysplastic cells and marked surface overexpression in OE33, OE19, and FLO-1 EAC cells. CKAP4 depletion reduced CKAP4 mRNA and protein, altered cell morphology, decreased viability and proliferation, and increased apoptosis. AKT phosphorylation was reduced in OE19 cells and MAPK phosphorylation in OE33 cells, whereas ß-catenin and GSK3ß activity remained unchanged. CKAP4 silencing increased surface expression of adhesion molecules, including integrins α5/ß1 in OE33 cells. RNA sequencing identified >500 differentially expressed genes enriched in immune-related pathways, indicating immune-related transcriptional changes.

**Conclusion:**

CKAP4 is a functionally relevant, surface-overexpressed protein in Barrett’s-associated EAC. These findings define cellular and transcriptional changes associated with CKAP4 depletion in EAC cells in vitro and support further mechanistic and preclinical investigation; therapeutic and biomarker relevance in EAC remains to be established.

## Highlights

• CKAP4 surface expression differs along Barrett’s sequence.
• CKAP4 silencing reduces proliferation and induces apoptosis in EAC cell models.
• CKAP4 depletion alters proliferation, adhesion and cell viability.
• RNA-seq revealed alterations in immune-related transcriptional pathways following CKAP4 depletion.
• DKK1-CKAP4 signaling in EAC appears cell-line specific.

## Graphical abstract

**Figure:**
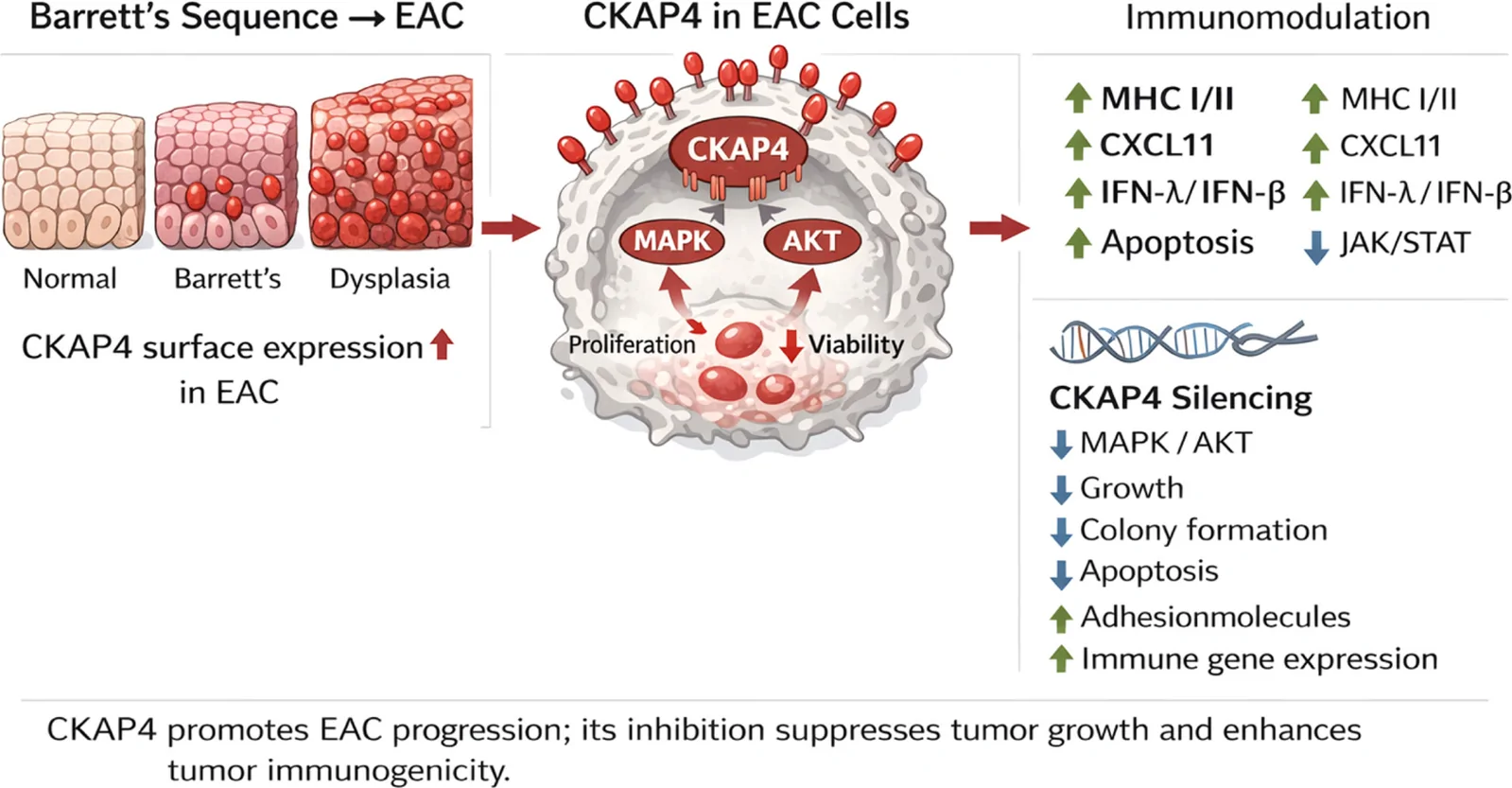

## Background

Esophageal cancer is histologically classified into two primary subtypes: esophageal squamous cell carcinoma (ESCC) and esophageal adenocarcinoma (EAC). While ESCC remains the predominant subtype globally, the incidence of EAC has increased substantially in Western countries over the recent decades [1], DOI: 10.1136/gutjnl-2012–302,412]. In Germany, adenocarcinomas accounted for 47% of all esophageal cancer cases in 2019, with a notable predominance in men [2]. That same year, approximately 5600 men and 1700 women were diagnosed with esophageal cancer in Germany [2], DOI: 10.18444]. Barrett’s esophagus (BE), a metaplastic transformation of the normal squamous epithelium to a columnar-lined epithelium with goblet cells, represents the most significant precursor lesion to EAC. This condition is strongly associated with chronic gastroesophageal reflux disease (GERD), along with other established risk factors, including age over 50, visceral obesity, tobacco use, a history of peptic strictures, and erosive esophagitis [3], PMCID: PMC9858189 [4], ID: NBK459330]. Although the progression from BE to EAC is relatively infrequent, patients with BE remain at elevated risk, particularly those with long-segment BE. The annual progression rate to EAC is estimated at 0.22% for long-segment BE, compared to 0.03% and 0.01% for short- and ultra-short-segment BE, respectively [5], PMCID: PMC1774141 [6], DOI: 10.1136/gutjnl-2015-309220]. Due to the often insidious onset of symptoms such as dysphagia, retrosternal pain, hoarseness, and unintentional weight loss, EAC is frequently diagnosed at a locally advanced stage. In such cases, a multimodal treatment approach combining surgical resection with chemotherapy and/or radiotherapy is the current standard. The treatment concepts for locally advanced EAC include perioperative chemotherapy with the FLOT protocol and neoadjuvant chemoradiotherapy following the CROSS regimen [7], DOI: 10.1097/SLA.0000000000005617]. However, the recently published ESOPEC trial indicated a significant survival advantage with the FLOT regimen, reporting a median overall survival (OS) of 66 months versus 37 months for CROSS [8], DOI: 10.1056/NEJMoa2409408]. Despite these advances, long-term outcomes for EAC remain dismal, with relative 10-year survival rates of 16–17% only [9], DOI: 10.1097/JTO.0b013e3182397751]. These statistics underline the critical need for improved understanding of the molecular mechanisms driving EAC progression to facilitate the development of novel diagnostic biomarkers and targeted therapies.

Cytoskeleton-associated protein 4 (CKAP4) is a type II transmembrane protein first identified in the early 1990s [10], DOI: 10.1242/jcs.104.3.671]. It plays a key structural role in anchoring and stabilizing the endoplasmic reticulum to the cytoskeleton [10,11], PMCID: PMC2120087 [12], DOI: 10.1016/j.cell.2010.11.011]. Beyond its structural function, CKAP4 also acts as a multifunctional receptor for several extracellular ligands. Notably, all members of the Dickkopf (DKK1-4) protein family can bind to CKAP4. Upon binding of DKK1, for example, the receptor is internalized, initiating downstream signaling through the phosphatidylinositol 3-kinase (PI3K)/AKT pathway and promoting cellular proliferation [13], PMCID: PMC4922689 [14], DOI: 10.1158/0008-5472.CAN-18-1749]. The biological functions of CKAP4 are context-dependent, varying with ligand interactions and the specific signaling pathways activated. Consequently, CKAP4 is implicated in both physiological cellular regulation and pathological processes, including cancer. CKAP4 has been implicated in the tumorigenesis of multiple cancer types, including pancreatic, lung, and renal cancers [13,15], DOI: 10.1158/1078-0432.CCR-18-2124, [16], PMCID: PMC5706004], as well as ESCC [17], DOI: 10.1038/s41388–018-0179-2]. In ESCC, the interaction of CKAP4 with its ligands, notably DKK1 and DKK3, promotes tumor cell proliferation through activation of the PI3K/AKT signaling pathway [14,17]. Shinno et al. demonstrated that co-expression of DKK1 and its receptor CKAP4 promotes PI3K/AKT-mediated proliferation in ESCC and is associated with poor prognosis, and may represent a therapeutic target, as anti-CKAP4 antibody treatment suppressed tumor growth in vitro and in xenograft models [17]. However, the role and underlying mechanisms of CKAP4-mediated cell proliferation in EAC remain poorly defined.

Alterations in the Wnt/ß-Catenin signaling pathway, including loss of negative regulators and aberrant activation, are thought to contribute significantly to the neoplastic progression from BE to EAC [18], DOI: 10.1038/modpathol.3880194 [19], DOI: 10.1111/j.1349-7006.2007.00663.x]. DKK1, a known antagonist of the Wnt-pathway, inhibits Wnt-induced ß-Catenin stabilization by binding to the extracellular domain of LRP6, a Wnt-co-receptor [20], DOI: 10.1038/nature756]. This inhibitory function supports the role of DKK1 as a tumor suppressor. However, paradoxically, DKK1 overexpression has been reported in several malignancies, such as hepatocellular carcinoma, multiple myeloma and urothelial carcinoma, where it correlates with poorer patient survival, suggesting a tumor-promoting role [21], PMCID: PMC4963804 [22], DOI: 10.1007/s13277-013-1411-x]. Consistent with this, elevated DKK1 expression has been observed in dysplastic BE and particularly in EAC [23], DOI: 10.1007/s00428-009-0769-z [24], PMCID: PMC4547437]. Recent studies by Lyros et al. [25]. PMCID: PMC6405970] demonstrated that DKK1 enhances tumor cell proliferation and invasion in EAC via mechanisms independent of canonical Wnt/ß-Catenin signaling. Instead, DKK1 activates AKT-mediated intracellular pathways and disrupts negative regulation of the Wnt-axis. Furthermore, the tumorigenic effects of DKK1 overexpression are at least partly mediated through its interaction with CKAP4.

Based on current evidence, DKK1 is proposed to act as a tumor promoter in EAC through binding to a specific receptor that activates AKT signaling. In our current study, we aimed to identify CKAP4 as this receptor and to elucidate its role in tumor progression by using in vitro models of esophageal cancer cell lines. Through targeted silencing of CKAP4, we investigated its impact on cancer cell proliferation, viability, adhesion, and key signaling pathways. Additionally, we investigated whether CKAP4 is associated with DKK1-related signaling and cellular phenotypes in EAC in vitro. Conjointly, our analyses were intended to provide novel insights into the functional role of CKAP4 in EAC tumor biology.

## Material and methods

### Cell culture

Eight human esophageal cell lines representing distinct stages of BE-associated cancer progression, from normal squamous epithelium to EAC, were utilized. Immortalized human esophageal squamous epithelial cell lines EPC1-hTERT (EPC1) and EPC2-hTERT (EPC2), kindly provided by Dr. Hiroshi Nakagawa (Gastroenterology Division, University of Pennsylvania, Philadelphia, PA, USA), were maintained in keratinocyte serum-free medium (KSFM; Thermo Fisher Scientific, Darmstadt, Germany) supplemented with 50 µg/mL bovine pituitary extract, 1 ng/mL recombinant human EGF (ImmunoTools, Friesoythe, Germany), and 1% penicillin-streptomycin (Thermo Fisher Scientific). The metaplastic cell line CP-A (CRL-4027) and the high-grade dysplastic cell line CP-B (CRL-4028) were obtained from LGC Standards (Wesel, Germany) and cultured in MCDB-153 medium (Biochrom, Berlin, Germany) supplemented as per the manufacturer’s instructions. EAC cell lines OE33 (ECACC 96070808), OE19 (ECACC 96071721), FLO-1 (ECACC 11012001), and SK-GT-4 (ECACC 11012007) were purchased from Sigma-Aldrich (Taufkirchen, Germany). OE33, OE19, and SK-GT-4 cells were cultured in RPMI 1640 medium (Gibco, Waltham, USA) supplemented with 10% fetal bovine serum (FBS; Biochrom), while FLO-1 cells were maintained in DMEM supplemented with 2 mM glutamine and 10% FBS. All cell lines were cultured at 37 °C in a humidified atmosphere with 5% CO₂.

### CKAP4 gene silencing

OE33 and OE19 cells were transfected with MISSION® endoribonuclease-prepared small interfering RNAs (siRNAs), specifically targeting human CKAP4 (EHU063061, Sigma-Aldrich, Taufkirchen, Germany), which are a heterogeneous pool of multiple siRNA species generated from a broader target region. Non-targeting siRNAs (NT-siRNA; SIC001, Sigma-Aldrich, Taufkirchen, Germany) served as negative controls, while untreated cells were included as mock controls (MC). Transfections were carried out using Opti-MEM medium, Lipofectamine 2000, and Plus Reagent (Thermo Fisher Scientific, Darmstadt, Germany) according to the manufacturer’s instructions, with a final siRNA concentration of 25 nM.

### RNA isolation and real-time quantitative polymerase chain reaction (qPCR) analysis

Total RNA was isolated from cultured cells using the RNeasy Plus Mini Kit (Qiagen, Hilden, Germany) according to the manufacturer's instructions. Complementary DNA (cDNA) was synthesized from 250–1000 ng of total RNA using the RevertAid First Strand cDNA Synthesis Kit (Thermo Fisher Scientific, Darmstadt, Germany). Quantitative real-time PCR (qRT-PCR) was performed using the SYBR Green Master Mix (Thermo Fisher Scientific, Darmstadt, Germany) and analyzed on a LightCycler 480 System (Roche Diagnostics GmbH, Mannheim, Germany). Gene expression levels were quantified using the ΔΔCt method, with ß-actin serving as the reference gene. Primer sequences used for qRT-PCR are provided in Table 1.

**Table 1:** Oligo sequences of primers used for qRT-PCR and antibodies used for Western blot analysis.

**Gene**	**Forward**	**Reverse**	**Length (bp)**
ß-actin	GTCTTCCCCTCCATCGTG	AGGGTGAGGATGCCTCTCTT	113
CKAP4	TCTCAGGTCTGAGGAGTCCG	CCTCAAAGGCTTCCGAGTGT	134
DKK1	AGCGTTGTTACTGTGGAGAAG	GTGTGAAGCCTAGAAGAATTACTG	82
Ki-67	TGACTTCCTTCCATTCTGAAGAC	TGGGTCTGTTATTGATGAGCC	109
p21	GGA AGA CCA TGT GGA CCT GT	GGCGTTTGGAGTGGTAGAAA	146
BCL2	GAGAAATCAAACAGAGGCCG	CTGAGTACCTGAACCGGCA	106
	**Unique Assay ID**	**Catalogue number**	
CX3CL1	qHsaCEP0049180	10031225	
INHBA	qHsaCIP0027657	10031225	

	**Dilution**	**Company**	**Catalogue number**
**Primary antibodies**			
CKAP4	1:1000 (1% BSA / TBST)	Abcam, Cambridge, UK	ab223774
ß-actin	1:10.000 (1% BSA / TBST)	Sigma-Aldrich, Taufkirchen, Germany	A1978
DKK1	1:5000 (0,5% LFM / TBST)	Abcam, Cambridge UK	ab109416
p-GSK3ß-Ser9	1:1000 (5% BSA / TBST)	Cell Signaling Technology, Danvers, USA	5558
GSK3ß	1:1000 (5% BSA / TBST)	Cell Signaling Technology, Danvers, USA	12456
p-AKT-Ser473	1:2000 (5% BSA / TBST)	Cell Signaling Technology, Danvers, USA	4060
AKT	1:1000 (5% BSA / TBST)	Cell Signaling Technology, Danvers, USA	4691
p-MAPK- Thr202/Tyr204	1:2000 (5% BSA / TBST)	Cell Signaling Technology, Danvers, USA	4370
MAPK	1:1000 (5% BSA / TBST)	Cell Signaling Technology, Danvers, USA	4695
p-ß-Catenin-Ser552	1:1000 (5% BSA / TBST)	Cell Signaling Technology, Danvers, USA	5651
ß-Catenin	1:1000 (5% BSA / TBST)	Cell Signaling Technology, Danvers, USA	8480
H3 (pan)	1:5000 (5% BSA / TBST)	Cell Signaling Technology, Danvers, USA	4499
GAPDH	1:5000 (0.5% LFM / TBST)	Thermo Fisher Scientific, Waltham, USA	39–8600
**Secondary antibodies**			
Goat Anti-rabbit	1:7500 (0.5% LFM / TBST)	Jackson Immuno Research, Suffolk, UK	111–035–045
Goat Anti-mouse	1:7500 (0.5% LFM / TBST)	Jackson Immuno Research, Suffolk, UK	115–035–068

BSA: bovine serum albumin; LFM: low fat milk; TBST: Tris-buffered saline with 0.1% (v/v) Tween 20.

### Western blot analysis

Following cell harvesting, lysis was performed in 100 µL of RIPA buffer supplemented with protease and phosphatase inhibitors (Sigma-Aldrich, Taufkirchen, Germany). Protein concentrations were determined using the Bradford assay. Equal amounts of protein (10–20 µg) were separated on 10% SDS-polyacrylamide gels and transferred to nitrocellulose membranes using a fast semi-dry transfer system. Transfer efficiency was verified by Ponceau S staining. Membranes were blocked in 5% low-fat milk (Carl Roth, Karlsruhe, Germany) in Tris-buffered saline containing 0.1% (v/v) Tween-20 (TBST) for 1 h at room temperature, followed by overnight incubation at 4 °C with the appropriate primary antibodies. Subsequently, membranes were incubated with horseradish peroxidase (HRP)-conjugated goat anti-rabbit or goat anti-mouse secondary antibodies for 1 h at room temperature. Signal detection was performed using an enhanced chemiluminescence (ECL) kit (Millipore, Billerica, USA). For reprobing, membranes were stripped using Roti®-Free Stripping Buffer 2.0 (Carl Roth, Karlsruhe, Germany) for 1 h. Antibodies and dilutions used are listed in Table 1. Densitometric analysis of protein bands was carried out using ImageJ software (https://doi.org/10.1038/nmeth.2089).

### Microscopic image

Morphological changes were assessed by phase-contrast microscopy in OE33 and OE19 cells at 24 and 48 h after transfection with CKAP4-targeting siRNA. Representative images were recorded to document changes in cell morphology and cell density.

### Cellular assays

Cell viability and proliferation were assessed using the PrestoBlue™ Cell Viability Reagent (Thermo Fisher Scientific, Darmstadt, Germany). OE33 and OE19 cells were seeded at a density of 5000 cells per well in 96-well plates and cultured in complete medium. After 24 h, the medium was removed, and CKAP4 gene silencing was performed as described above. Each well contained 90 µL of culture medium. At 24, 48, and 72 h post-transfection, 10 µL of PrestoBlue reagent were added per well, followed by incubation at 37 °C for 10 min. Fluorescence was measured at 560 nm excitation using a SpectraMax M5 plate reader (Molecular Devices, Sunnyvale, USA). Non-transfected cells served as negative controls.

For the colony formation assay, which evaluates the long-term proliferative capacity of cells, OE33 and OE19 cells were transfected with CKAP4-siRNA as described above. Forty-eight hours post-transfection, cells were trypsinized, and 500 cells per condition were seeded into 6-well plates containing RPMI medium. Cells were incubated without medium change for 10 days to allow colony formation. Colonies were fixed and stained with crystal violet solution, and the number of colonies was quantified by counting using ImageJ software.

### Flow cytometry

Cell surface expression of CKAP4 was analyzed across all cell lines representing the BE sequence using a CKAP4-specific antibody (bs-6502R-A647, Bioss Inc., Boston, USA). To evaluate the impact of gene silencing, CKAP4 surface expression was also assessed following siRNA treatment. Apoptosis following CKAP4-targeted siRNA treatment was detected using the FITC Annexin V Apoptosis Detection Kit with PI (BioLegend, San Diego, USA), following the manufacturer’s instructions. To investigate the role of CKAP4 in cell adhesion, expression levels of selected cell surface receptors were quantified post-knockdown using a BD LSRFortessa™ Cell Analyzer (BD Biosciences, Franklin Lakes, NJ, USA). Cell surface expression of CKAP4 was analyzed by indirect immunofluorescence flow cytometry using a rabbit anti-CKAP4 primary antibody and a fluorophore-conjugated goat anti-rabbit secondary antibody. Adhesion molecule expression was assessed by multiparameter flow cytometry using fluorophore-conjugated monoclonal antibodies. Details of the antibodies and dilutions used for flow cytometry are provided in Table 2.

**Table 2:** Antibodies and dilutions used for Flow cytometry analysis.

	**Conjugation**	**Dilution**	**Company**	**Catalogue number**
CKAP4	AF647	1:100	Bioss Inc, Boston, USA	bs-6502R-A647
	AF488	1:100	Bioss Inc, Boston, USA	bs-6520R-A488
MUC1 / CD227	APC	1:100	BioLegend, San Diego, USA	355608
SELPLG / CD162	BV421	1:100	BioLegend, San Diego, USA	328808
NCAM-1 / CD56	BV650	1:100	BioLegend, San Diego, USA	362531
CD44	BV785	1:200	BioLegend, San Diego, USA	103041
CDH2 / CD325	AF488	1:100	BioLegend, San Diego, USA	350810
ICAM-3 / CD50	PerCP Cy5.5	1:100	BioLegend, San Diego, USA	330005
ADGRG1 / GPR56	PE-Cy7	1:200	BioLegend, San Diego, USA	358205
EPCAM / CD326	BV605	1:200	BioLegend, San Diego, USA	324208
ALCAM / CD166	BUV395	1:400	BioLegend, San Diego, USA	343909
PECAM-1 / CD31	BV711	1:100	BioLegend, San Diego, USA	303124
ICAM-1 / CD54	BUV737	1:200	BioLegend, San Diego, USA	353106
ICAM-2 / CD102	PE	1:200	BioLegend, San Diego, USA	328507
CDH1 / CD324	PE-Dz	1:400	BioLegend, San Diego, USA	324115
α5 Integrin / CD49e	PE	1:100	BioLegend, San Diego, USA	328009
IgG2, Isotype Ctrl	PE	1:100	BioLegend, San Diego, USA	401207
ß1 Integrin / CD29	APC	1:100	BioLegend, San Diego, USA	303007
IgG1, Isotype Ctrl	APC	1:100	BioLegend, San Diego, USA	400119
Rabbit IgG Isotype Control	AF647	1:100	Bioss Inc, Boston, USA	bs-0295P-A647
Rabbit IgG Isotype Control	AF488	1:100	Bioss Inc., Boston, USA	bs-0295P-A488

### RNA sequencing

For sequencing, siCKAP4 OE33 cells were lysed, centrifuged and fixed in liquid nitrogen. Transfection was done for 48 h. RNA isolation, library preparation and sequencing were done by *GENOWITZ Leipzig GmbH* (Azenta Life Sciences, Leipzig, Germany). As all samples were processed and sequenced under identical experimental conditions, no batch correction was required.

Differential gene expression analysis, as well as gene ontology and pathway enrichment analyses, were performed to compare siNT- and siCKAP4-treated OE33 cells. Here, differentially expressed genes with an adjusted p-value <0.05 and an absolute log2-fold-change greater than 1 were further analyzed. The functional characterization as well as selection was done with Webgestalt (webgestalt.org). The visualization of the affected genes within the selected pathways was performed within R (r-project.org) using the package ‘Pathview’ provided by Bioconductor (bioconductor.org). The objective was to identify specific signal transduction pathways and associated key regulatory molecules significantly associated with CKAP4-targeting siRNA-mediated depletion.

### Analysis of publicly available transcriptomic datasets and published clinical tissue data

To complement the experimental findings, we assessed CKAP4 expression in two independent esophageal adenocarcinoma datasets. For the GEO analysis, we used the normalized microarray dataset GSE13898, which includes primary esophageal adenocarcinoma tissues and surrounding normal tissues. According to the GEO record, GSE13898 contains 64 primary esophageal adenocarcinoma samples, and 28 surrounding normal fresh-frozen tissues. For the TCGA analysis, RNA-seq expression data and corresponding clinical information were obtained from the TCGA esophageal carcinoma cohort (TCGA-ESCA), and only histologically annotated esophageal adenocarcinoma cases were retained for analysis. For differential expression analysis, CKAP4 expression levels were compared between tumor and normal tissues using the Wilcoxon rank-sum test. All analyses were performed using R.

### Statistical analysis

Calculation was performed using GraphPad Prism 10 (GraphPad Software Inc, San Diego, USA). All data were presented as mean ± standard deviation. A *p*-value of <0.05 was considered statistically significant.

## Results

### CKAP4 is overexpressed at the surface of BE-associated EAC cells

To investigate CKAP4 and its functional role, it was essential to confirm its expression in the cell lines studied. We demonstrated that CKAP4 expression varied along Barrett’s sequence. Total mRNA levels were quantified by qPCR (Fig. 1A). Surface expression of CKAP4 was investigated with flow cytometry analysis (Fig. 1B), whereas protein expression was assessed via Western blot analysis (Fig. 1C, D). Both mRNA and protein expression showed a trend toward reduced levels in malignant cells, with the lowest expression observed in SK-GT-4 cells. In contrast, the dysplastic cell line CP-B exhibited the highest CKAP4 expression. Notably, flow cytometry analysis revealed significant higher CKAP4 cell surface expression in the carcinoma cell lines OE33, OE19, and FLO-1 relative to EPC1, indicating elevated receptor presence in these cells.

**Fig. 1:**
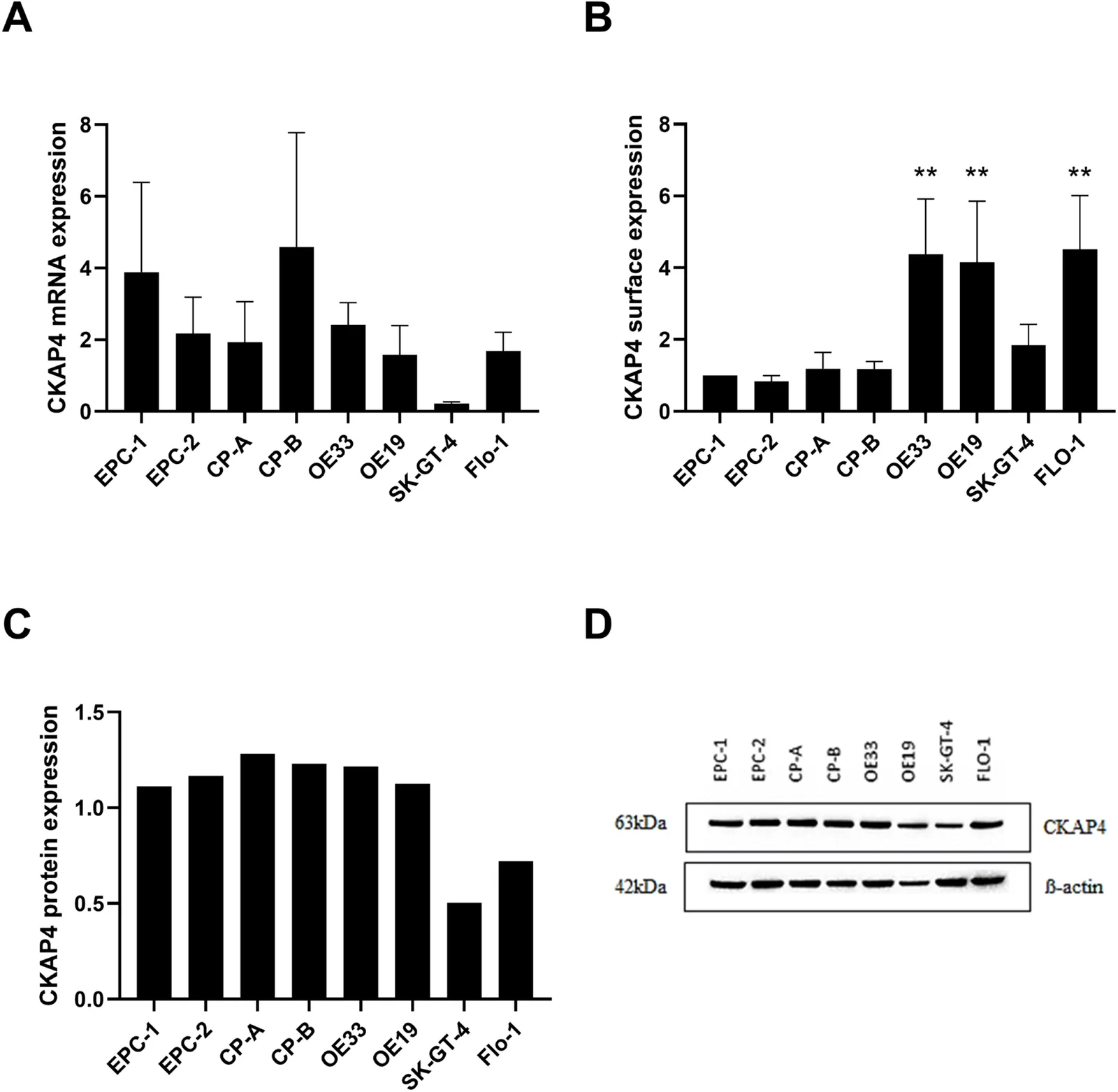
Expression of CKAP4 along Barrett´s sequence. Total mRNA (A), surface (B) and protein (C, D) expression of CKAP4 was detected in EPC-1 and EPC-2 (esophageal squamous epithelium), CP-A (metaplastic epithelium), CP-B (high-grade dysplastic epithelium) and OE33, OE19, FLO-1 and SK-GT-4 (esophageal adenocarcinoma) cells. Real-Time PCR and Western blot analysis show differential CKAP4 expression with lower levels in SK-GT4 and FLO-1. As loading control serves ß-actin. A significantly higher surface expression of CKAP4 in cell lines OE33, OE19 and FLO-1 was detected by flow cytometry relative to EPC-1. Values are shown as mean ± SD (*p < 0.05; **p < 0.01; one-way ANOVA followed by Dunnett´s multiple comparisons test; n = 3).

### Differential CKAP4 expression between normal and tumor tissues in public clinical cohorts

Analysis of the GEO dataset (GSE13898) demonstrated significantly decreased CKAP4 expression in esophageal tumor tissue compared with normal controls. In contrast, analysis of the TCGA-ESCA cohort did not reveal a statistically significant difference in CKAP4 expression between tumor and non-tumor tissues (Supplemental Fig. 1 A, B).

### Efficient siRNA-mediated CKAP4 depletion in EAC cells

To investigate the role of CKAP4 in EAC tumor biology, we achieved efficient knockdown of CKAP4 in OE33 and OE19 cell lines. Cells were transfected with CKAP4-specific small interfering RNA (siCKAP4) for 48 (Fig. 2A) and 72 h (Fig. 2B), resulting in a significant reduction of CKAP4 mRNA expression as measured by qPCR. Knockdown efficiency was further validated at the protein level, with Western blot analysis confirming decreased CKAP4 protein abundance in both cell lines following siCKAP4 treatment (Fig. 2C–F). Microscopically, changes in morphology and a reduction in cell size could be observed as early as 24 h after transfection, alongside a higher proportion of apoptotic cells. These effects appeared to be even more pronounced after 48 h. (Fig. 3A–D). Quantitative analyses were performed to confirm this.

**Fig. 2:**
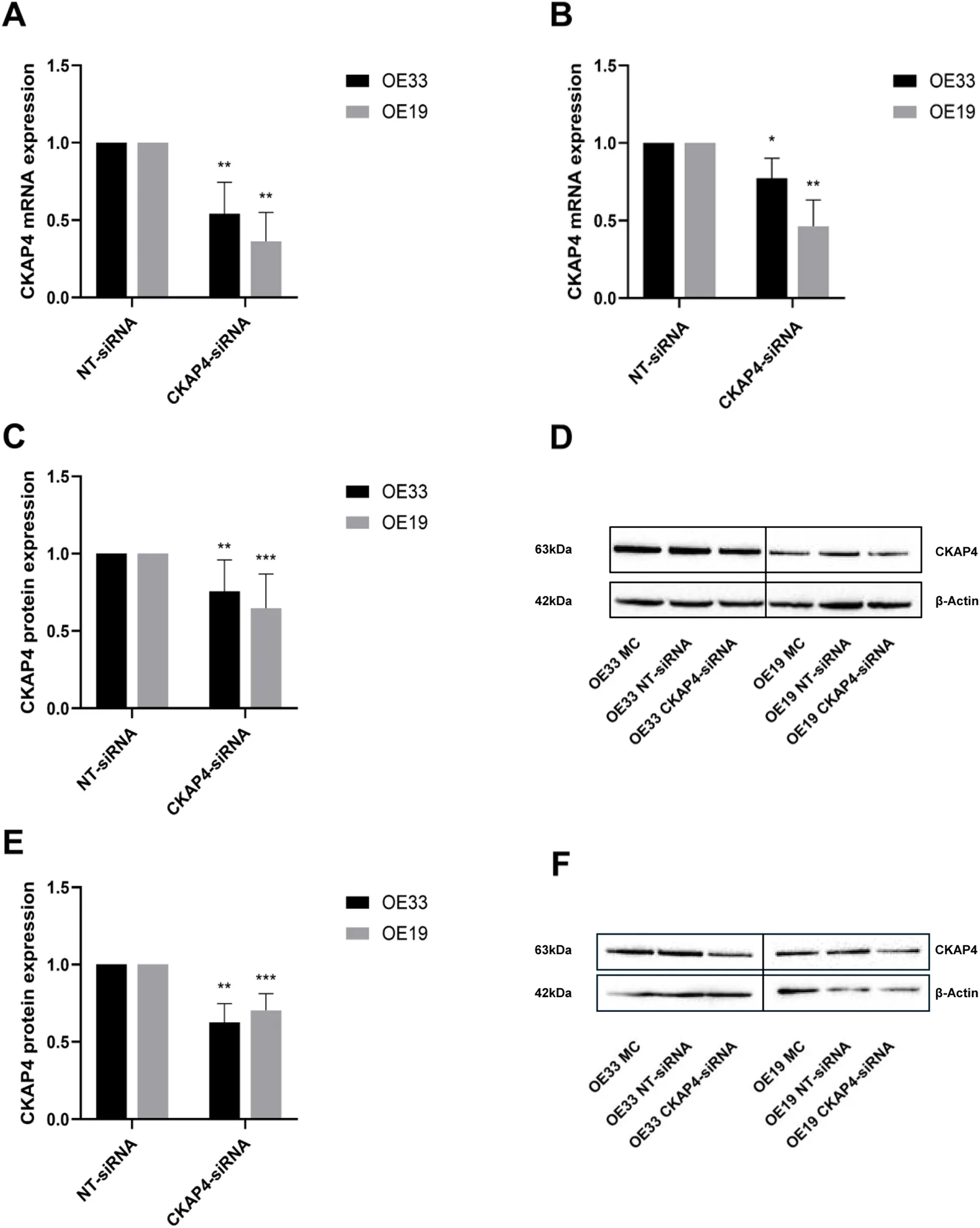
CKAP4 expression in OE33 and OE19 cells undergoing siRNA-mediated CKAP4 gene silencing. CKAP4-mRNA expression in OE33 and OE19 cells following CKAP4-siRNA transfection for 48 h (A) and 72 h (B), normalized to ß-actin. Specific knockdown of CKAP4 protein level in OE33 and OE19 cells, shown by Western blot analysis 48 h post-transfection (C, D). Western blot analysis demonstrates reduction of CKAP4 in OE33 and OE19 cells following siRNA-mediated CKAP4 gene silencing for 72 h (E, F). Loading control is ß-actin. Values are presented as mean ± SD and relative to NT-siRNA cells (NT; 1) (*p < 0.05; **p < 0.01; ***p < 0.001; *t*-test; n = 4).

**Fig. 3:**
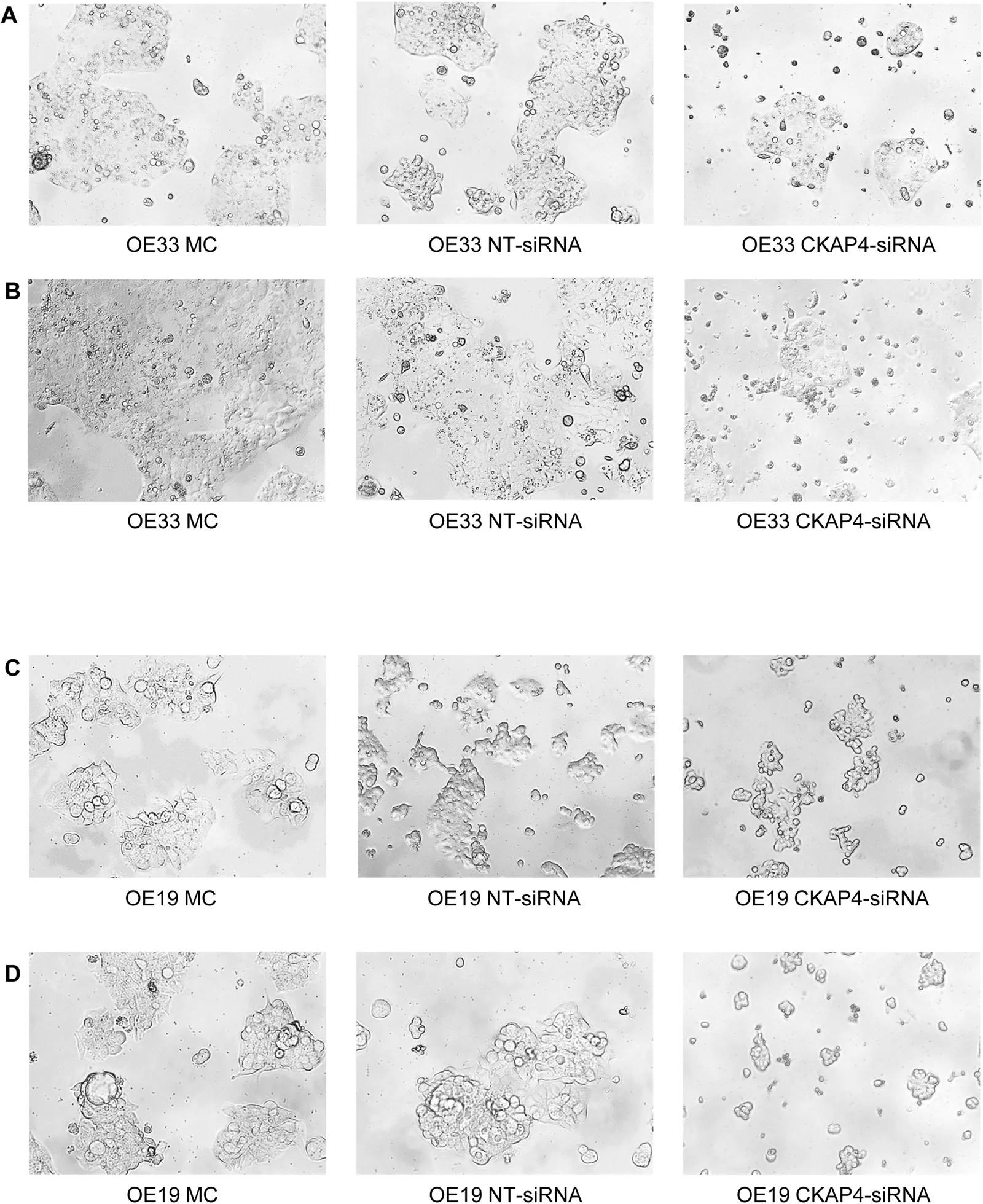
Microscopy of Mock Control (MC) and NT-siRNA as well as CKAP4-siRNA transfected OE33 cells, after siRNA-mediated CKAP4 gene silencing for 24 h (A) and 48 h (B). Mock, NT-siRNA and CKAP4-siRNA OE19 cells after 24 h (C) and 48 h (D) transfection time.

### DKK1 expression following CKAP4 depletion

To determine whether CKAP4 depletion was accompanied by changes in DKK1 expression, DKK1 mRNA and protein levels were assessed in OE33 and OE19 cells following treatment with CKAP4-targeting siRNA. After 48 h, DKK1 mRNA expression was increased in both cell lines. At 72 h, increased DKK1 mRNA expression persisted in OE33 cells but was no longer observed in OE19 cells (Fig. 4A). In contrast, DKK1 protein levels remained unchanged in both cell lines at the analyzed time points (Fig. 4B).

**Fig. 4:**
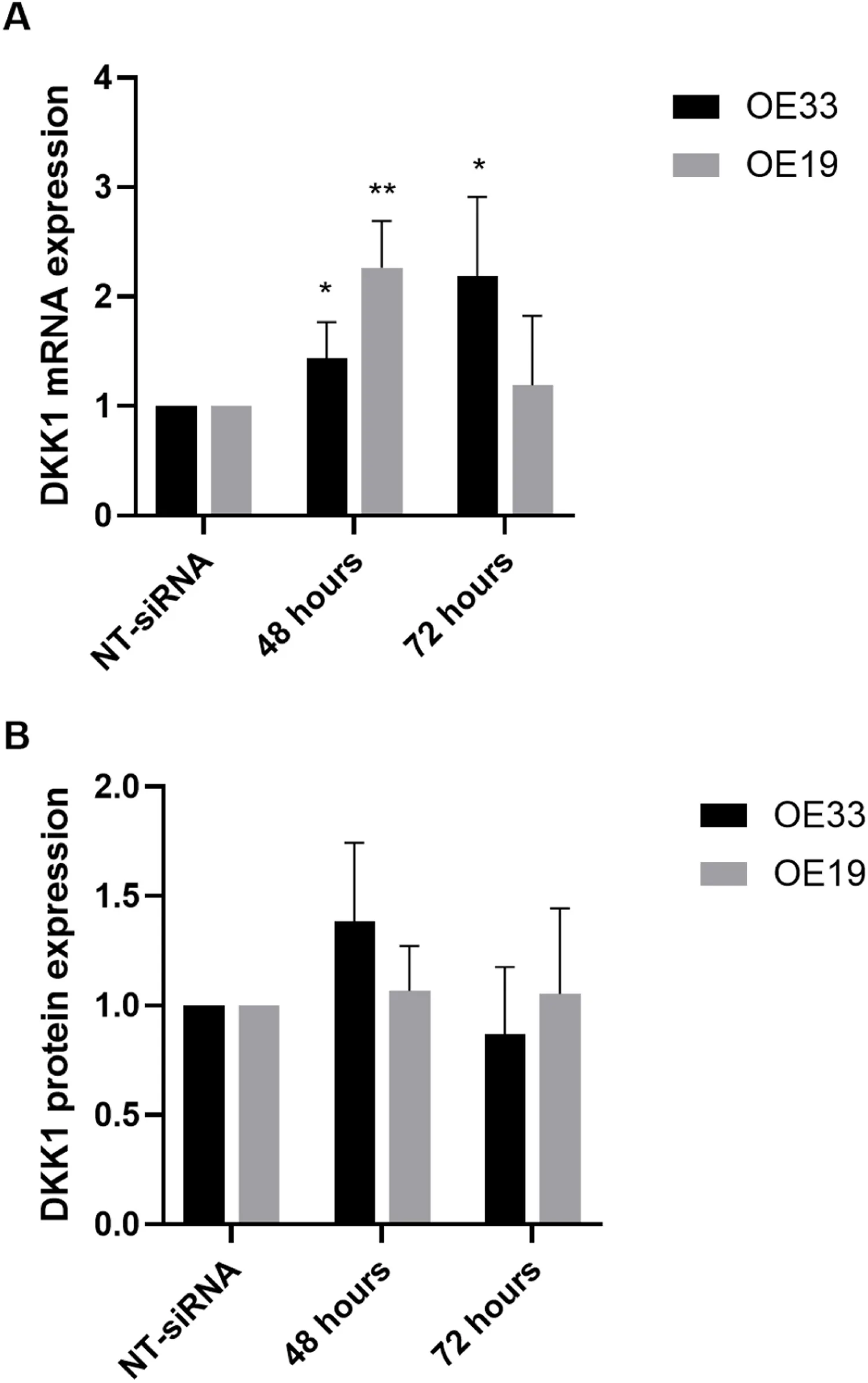
Quantitative RT-PCR demonstrates elevated DKK1-mRNA expression after siRNA-mediated CKAP4 gene silencing for 48 h and 72 h (A). DKK1 protein expression in OE33 and OE19 cells following transfection with CKAP4-siRNA for 48 h and 72 h (B). Values are shown as mean ± SD and relative to NT-siRNA cells (NT; 1) (*p < 0.05; *t*-test; n = 3).

### Phosphorylation of AKT, ß-Catenin, GSK3ß and MAPK after CKAP4-siRNA transfection

OE33 and OE19 cells were transfected with CKAP4-siRNA for 48 h, and successful knockdown was confirmed prior to analysis. Western blotting was used to assess phosphorylation levels of key signaling proteins, including AKT (Ser473), ß-Catenin (Ser552), GSK3ß (Ser9), and MAPK (Thr202/Tyr204). CKAP4 depletion resulted in decreased AKT phosphorylation in OE19 cells, while MAPK phosphorylation was significantly reduced in OE33 cells. No changes were observed in the phosphorylation status of ß-Catenin or GSK3ß following CKAP4 silencing. Furthermore, nuclear accumulation of ß-Catenin was not detected after CKAP4-siRNA treatment (Supplemental Fig. 2).

### CKAP4-targeting siRNA treatment is associated with reduced viability and colony formation in EAC cells

In OE33 and OE19 cells, the effects of CKAP4-targeting siRNA treatment on cell viability were assessed using the PrestoBlue Viability Reagent. CKAP4-targeting siRNA treatment was associated with a significant reduction in cell viability in both cell lines, with the magnitude of the reduction increasing over time (Fig. 5A). To assess longer-term proliferative capacity, colony formation assays were performed. In OE33 cells, CKAP4-targeting siRNA treatment significantly reduced both colony number and colony size, whereas no significant effect on colony formation was observed in OE19 cells (Fig. 5C, D). These findings indicate that CKAP4-targeting siRNA treatment is associated with reduced viability in both EAC cell lines and reduced colony-forming capacity in OE33 cells.

**Fig. 5:**
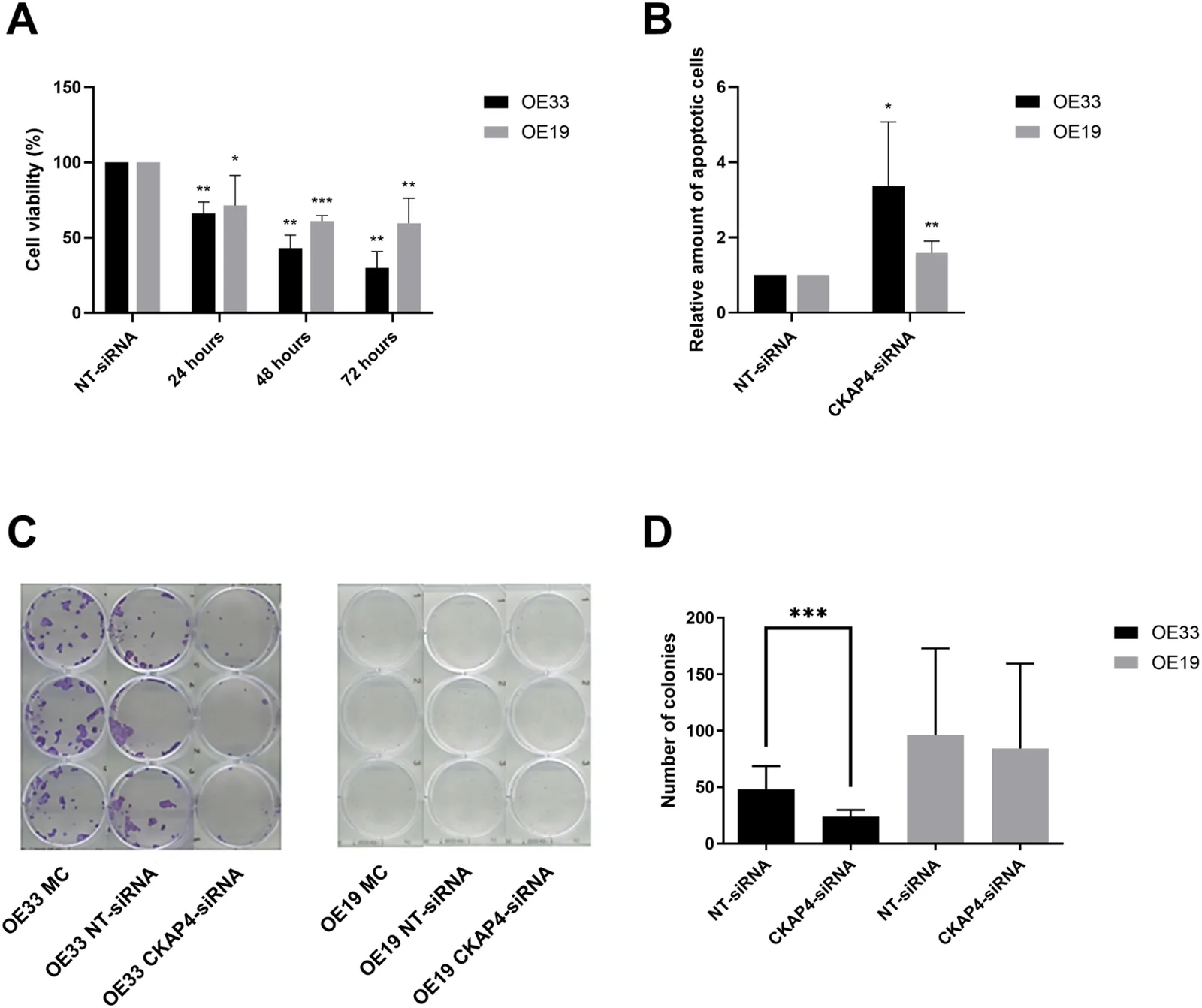
CKAP4 effects cell proliferation, viability and survival in OE33 and OE19 cells. Cell viability and proliferation assay in OE33 and OE19 cells after siRNA-mediated CKAP4 gene silencing. Measurements were conducted 24, 48 and 72 h after siRNA transfection (A). Values are presented as percentage relative to NT-siRNA treated cells (NT;100%) (*p < 0.05; **p < 0.01; ***p < 0.001; *t*-test; n = 4). Apoptosis assay to detect apoptotic OE33 and OE19 cells 48 h after CKAP4-siRNA transfection (B). Values are presented relative to NT-siRNA cells (NT; 1) (*p < 0.05; **p < 0.01, *t*-test; n = 3). Colony formation assay of OE33 and OE19 undergoing knockdown of CKAP4. Cells were fixed and stained after being transfected for 48 h and then grown for 10 days in RPMI medium without additives. Example pictures show the fixed and stained colonies of OE33 and OE19 cells (C). Values of both cell lines are presented as the mean number of colonies ± SD, a *t*-test was performed between NT-siRNA and CKAP4-siRNA group (D) (***p < 0.001; *t*-test; n = 18).

### CKAP4-targeting siRNA treatment is associated with increased apoptosis in OE33 and OE19 cells

Apoptotic cell death was assessed in OE33 and OE19 cells following treatment with CKAP4-targeting siRNA using Annexin V and PI staining. CKAP4-targeting siRNA treatment was associated with a significant increase in the proportion of apoptotic cells compared with the non-targeting siRNA control, corresponding to an approximately two-fold increase in OE19 cells and a three-fold increase in OE33 cells (Fig. 5B). These findings indicate that CKAP4-targeting siRNA treatment is associated with increased apoptotic cell death in both EAC cell lines.

### CKAP4-targeting siRNA treatment is associated with altered surface expression of adhesion molecules

Cell-surface expression of selected adhesion molecules was assessed by flow cytometry in OE33 and OE19 cells following treatment with CKAP4-targeting siRNA. In OE33 cells, CKAP4-targeting siRNA treatment was associated with significantly increased surface expression of MUC1, CD44, EPCAM, ALCAM, ICAM-1, ICAM-2, and CDH1 (Fig. 6A). In OE19 cells, increased surface expression was observed for EPCAM and ALCAM (Fig. 6B). We additionally examined the surface expression of integrin α5 and ß1, which together form the α5ß1 fibronectin receptor [26], DOI: 10.1021/bi025752f]. CKAP4-targeting siRNA treatment was associated with significantly increased expression of integrin α5 and ß1 in OE33 cells (Fig. 6A), whereas no significant changes were detected in OE19 cells (Fig. 6B). Taken together, these findings indicate that CKAP4-targeting siRNA treatment is associated with cell-line-specific alterations in the surface expression of multiple adhesion-related proteins in EAC cells.

**Fig. 6:**
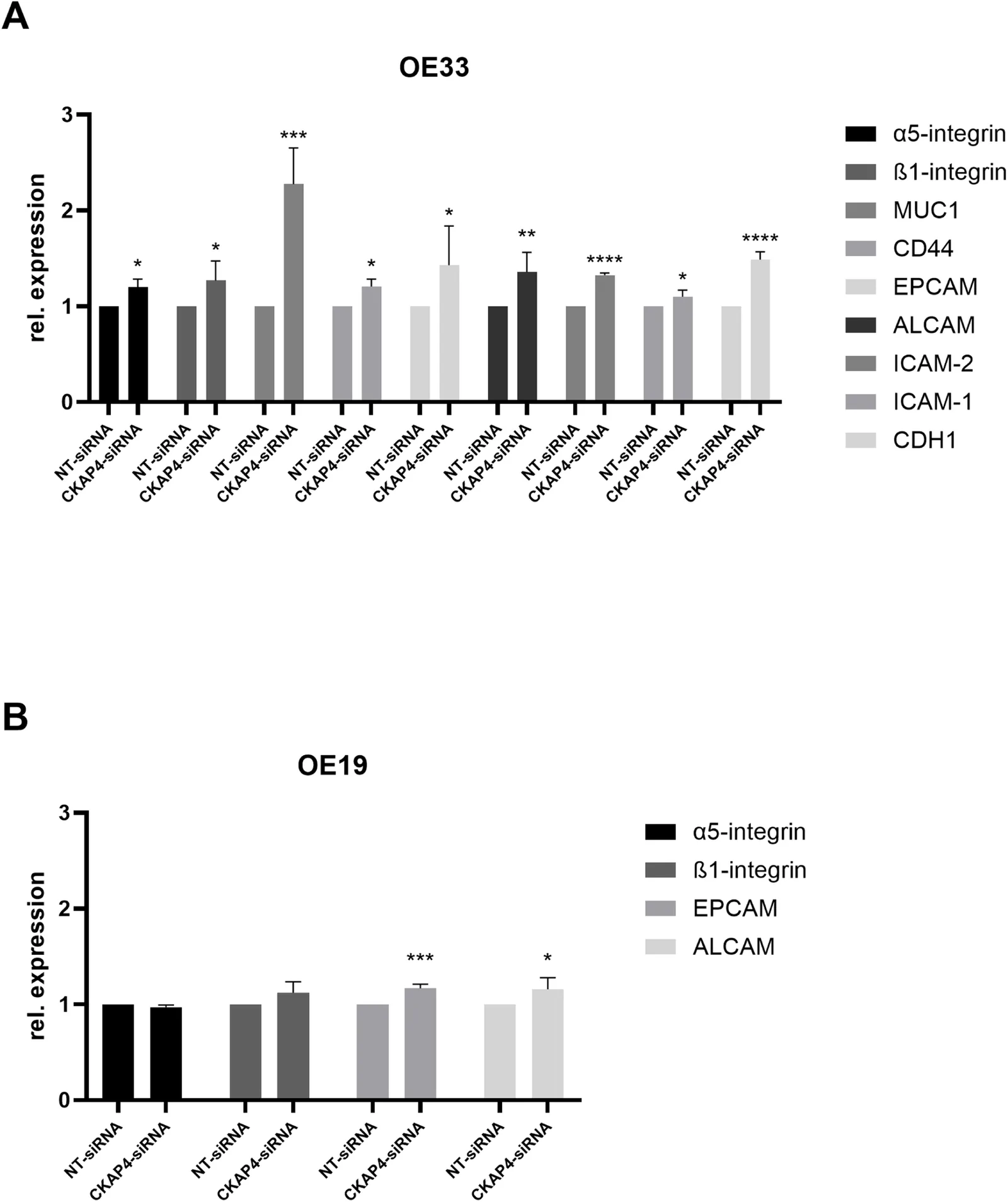
Expression of fibronectin receptor components α5 and ß1 integrin and further adhesion molecules at the cell membrane of OE33 (A) and OE19 (B) undergoing siRNA-mediated CKAP4 gene silencing. Values are presented as mean ± SD and relative to NT-siRNA cells (NT; 1) (*p < 0.05, **p < 0.01, ***p < 0.001, ****p < 0.0001, *t*-test; n = 4).

### Transcriptomic analysis reveals immune-related transcriptional changes following CKAP4 depletion in EAC cells

To investigate transcriptional changes associated with CKAP4 depletion, bulk RNA sequencing was performed in OE33 cells treated with either non-targeting siRNA or CKAP4-targeting siRNA. Approximately 500 genes were differentially expressed between the two conditions (Fig. 7A). KEGG pathway analysis identified significant alterations in pathways related to inflammatory and immune-associated signaling. Among the most strongly upregulated genes were CXCL11 and several interferons, including IFNL1, IFNL2, IFNL3, and IFNB1, whereas TNFSF18, TNFRSF10D, LEPR, IL1B, and ACVR2B were downregulated (Fig. 7B). CKAP4-targeting siRNA treatment was also associated with altered expression of genes involved in the JAK-STAT pathway (Fig. 8), as well as multiple interleukins and their receptors, CC and CXC chemokines, and components of TNFα- and NF-κB-associated signaling pathways. In addition, several genes involved in antigen processing and presentation, including MHC class I and class II molecules, were significantly upregulated following CKAP4-targeting siRNA treatment (Supplemental Fig. 3 A-G). Taken together, these findings indicate that CKAP4 depletion is associated with altered expression of genes involved in inflammatory, interferon, chemokine, and antigen-presentation pathways in OE33 cells.

**Fig. 7:**
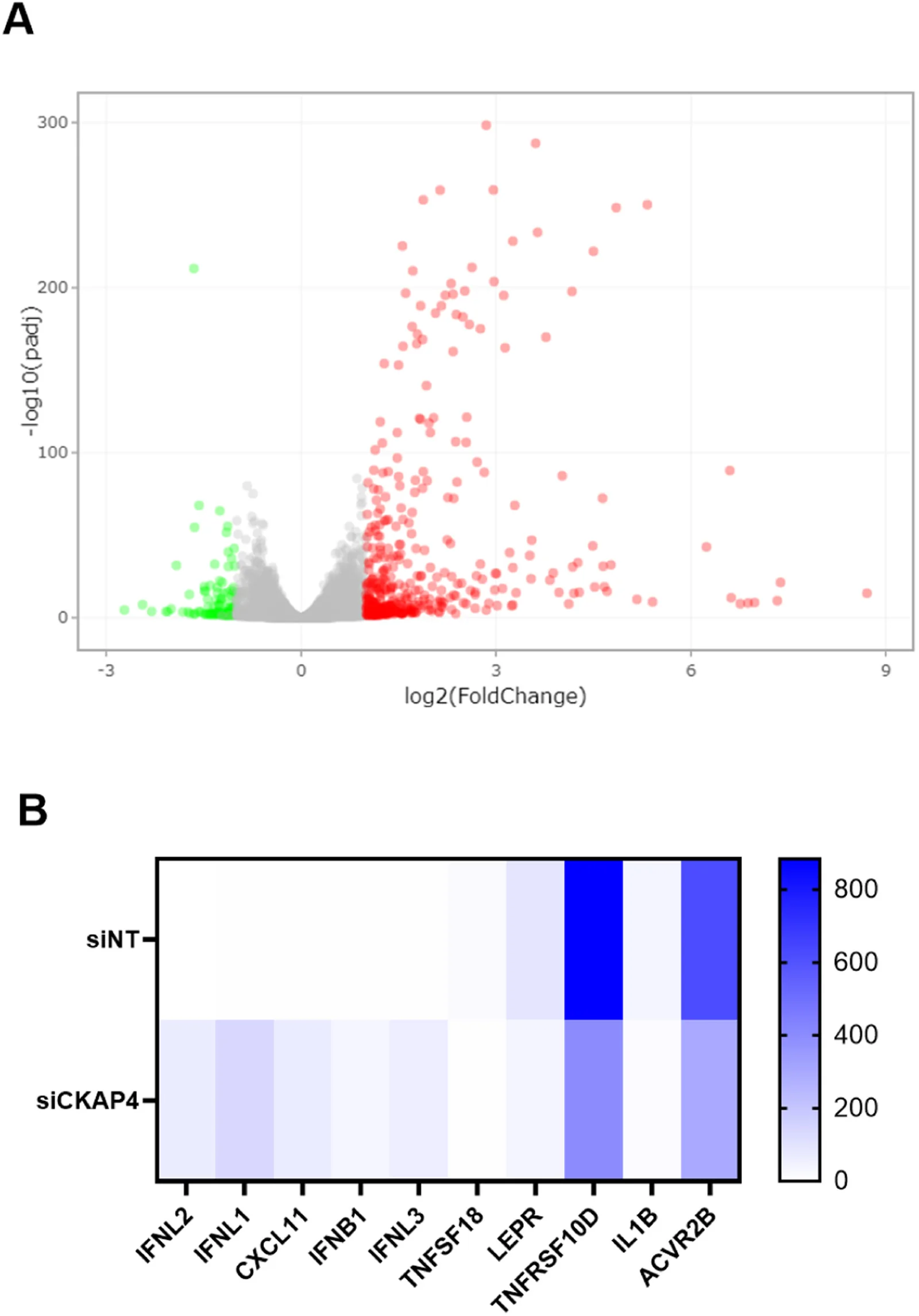
Differential gene expression of approximately 500 genes detected by bulk RNA sequencing comparing siNT versus siCKAP4 OE 33 knockdown cells (A; n = 3). Heatmap shows the five genes that were most strongly up- and downregulated after CKAP gene silencing (B).

**Fig. 8:**
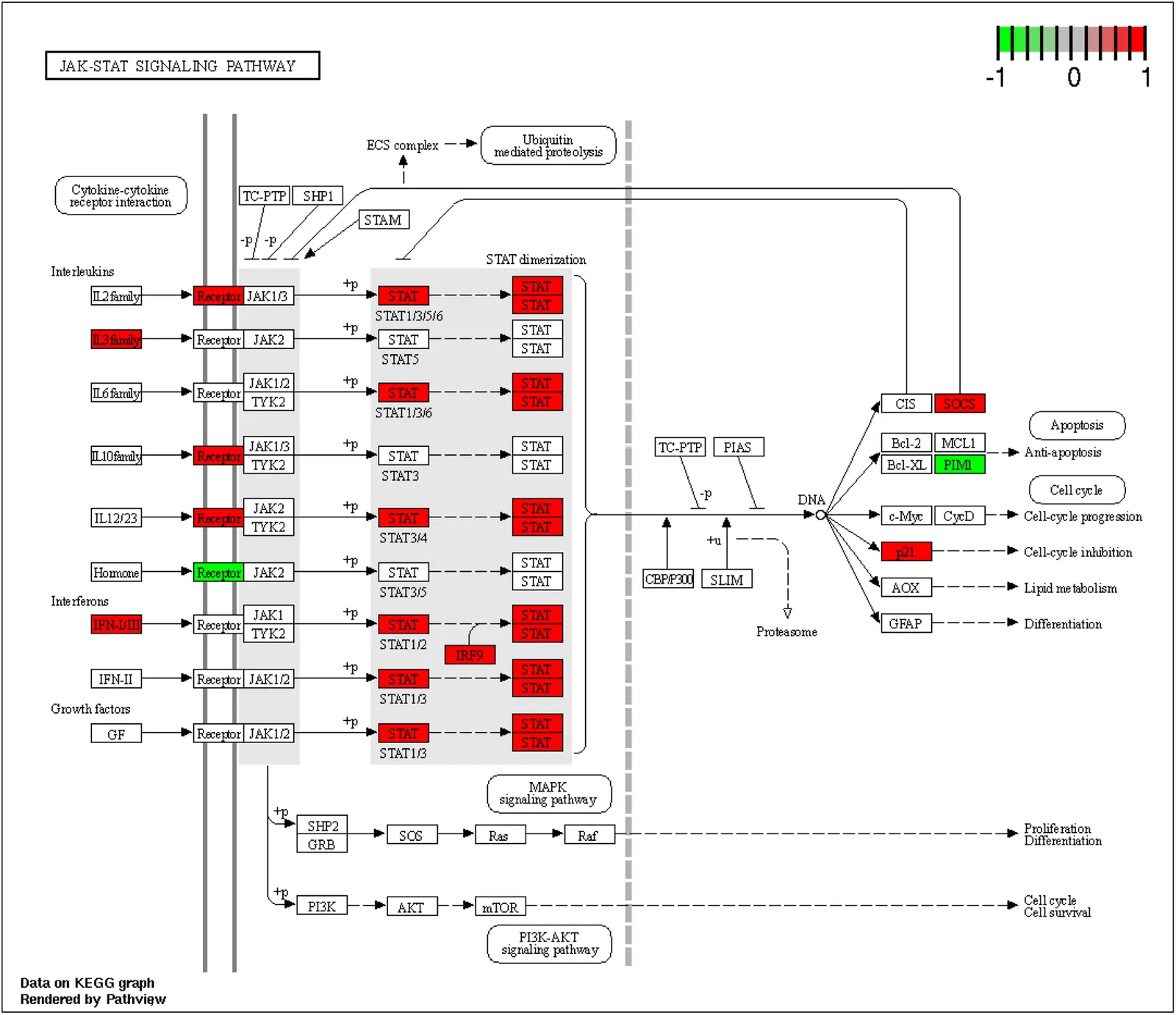
The impact of CKAP4 knockdown on the gene expression of JAK-STAT signaling pathway molecules was assessed using KEGG analysis. Values are presented relative to NT-siRNA cells (NT; 0; n = 3).

### Validation of RNA sequencing results by qPCR

To validate selected RNA-sequencing findings, the mRNA expression of p21, CX3CL1, BCL2, and INHBA was analyzed by quantitative real-time PCR. Consistent with the RNA-sequencing data, p21 and CX3CL1 were significantly upregulated following CKAP4-targeting siRNA treatment. In contrast, BCL2 and INHBA showed expression changes in the same direction as observed by RNA sequencing, but these differences did not reach statistical significance by qPCR (Supplemental Fig. 4).

## Discussion

In this study, we conducted a comprehensive cellular analysis of CKAP4 in EAC using an in vitro model encompassing cell lines representative of BE and EAC. Our data clearly demonstrated CKAP4 expression in all cell lines along the Barrett's sequence and confirmed CKAP4 localization as a cell surface receptor. Notably, CKAP4 surface expression was significantly higher in OE33, OE19 and FLO-1 than in the non-neoplastic EPC1 cell line. These results implied a potential association between CKAP4 surface expression and tumor development and progression.

Specific small interfering RNA transfection for 48 and 72 h achieved efficient CKAP4 knockdown in OE33 and OE19 cells, leading to significant reductions in CKAP4 mRNA and protein levels. Functional assays revealed that CKAP4 depletion significantly impaired cell proliferation, growth and viability. In line with these findings, CKAP4 silencing induced a marked increase in apoptosis, particularly in OE33 cells. The differential response observed between the two cell lines may be explained by variations in the DKK1-CKAP4 axis. OE19 cells exhibit minimal DKK1 expression [17], which has been shown to modulate CKAP4-dependent proliferation. These findings support an association between siRNA-mediated CKAP4 depletion and impaired growth phenotypes in EAC cells in vitro.

Notably, CKAP4 knockdown had a sustained inhibitory effect on cell viability. Viability declined progressively with longer transfection times. Colony formation assays corroborated this enduring effect, revealing that CKAP4-depleted OE33 cells exhibited a significant reduction in both colony number and size. These cells failed to recover growth even after ten days without siRNA exposure. Cell adhesion, which is a key factor in tumor metastasis, might also be affected by CKAP4 depletion. Significant upregulation of several adhesion molecules, including EPCAM and ALCAM, were observed in both OE33 and OE19 cells. EPCAM is a well-established epithelial cell adhesion molecule that is involved in cell-to-cell contact [27], DOI: 10.1083/jcb.125.2.437], while ALCAM contributes to adhesion and migration across various cell types [28], DOI: 10.1074/jbc.M407776200]. Both EPCAM and ALCAM are functionally relevant molecules that play an established role in cell-cell adhesion and epithelial integrity. Overexpression of CKAP4, which is particularly prevalent in EAC cell lines, leads to reduced EPCAM and ALCAM expression, resulting in increased tumor cell migration, which potentially can influence metastaticity . Furthermore, knockdown of CKAP4 in OE33 cells increased the expression of CDH1 (E-cadherin) and integrin α5ß1, which are both critical mediators of cell-cell and cell-matrix adhesion. CDH1 maintains epithelial integrity and is frequently downregulated during cancer progression and metastasis [29], DOI: 10.1074/jbc.M201984200]. The α5ß1 integrin fibronectin receptor plays a pivotal role in cell migration and extracellular matrix interactions [26]. Our findings suggest that CKAP4 depletion enhances fibronectin-dependent adhesion, which could counteract cell migration. These results are consistent with the work of Osugi et al. [30], PMCID: PMC6664606], who showed that CKAP4 controls cell adhesion and migration in pancreatic cancer by decreasing α5 integrin levels and preventing α5ß1 integrin recycling. Taken together, these data support a model in which CKAP4 might facilitates tumor metastasis by decreasing cell adhesion sites and promoting migratory behavior.

Flow cytometric analysis revealed increased surface expression of intercellular adhesion molecules ICAM-1 and ICAM-2 in OE33 cells following CKAP4 knockdown. ICAM-1, primarily expressed on epithelial cells, mediates lymphocyte binding and plays a critical role in diapedesis, a key step in leukocyte transmigration during inflammatory responses [31], PMCID: PMC7977775]. The elevated expression of ICAM-1 may be linked to the increased apoptosis observed upon CKAP4 silencing, potentially driven by cytokine signaling, including TNFα. ICAM-2 also contributes to immune cell trafficking by facilitating leukocyte migration, a fundamental process in immune surveillance and response [32], DOI: 10.1016/j.micpath.2017.01.021]. The upregulation of both ICAM-1 and ICAM-2 following CKAP4 knockdown suggests a role for CKAP4 in modulating immune-related signaling pathways, with potential implications for immune regulation within the tumor microenvironment.

RNA sequencing of OE33 cells following CKAP4-targeting siRNA treatment revealed a broad immune-related transcriptional signature. Genes involved in antigen processing and presentation, including several MHC class I and II molecules, were significantly upregulated [33], PMCID: PMC5159193]. In addition, multiple chemokines, cytokines, and their receptors were differentially expressed, including members of the CC and CXC chemokine families [34], DOI: 10.1016/j.arr.2023.101900 [35], PMCID: PMC6235799]. CKAP4 depletion was also associated with altered expression of components of the JAK-STAT, NF-κB, and NOD-like receptor pathways, indicating that CKAP4-associated cellular states may influence transcriptional programs linked to inflammatory and immune signaling. IL-1ß represented an exception to the general pattern of increased immune-related gene expression and was significantly downregulated following CKAP4 depletion; given the context-dependent roles of IL-1ß in inflammation and tumor biology, the functional significance of this change remains unclear [36], DOI: 10.1038/nrrheum.2010.4]. These findings are consistent with emerging evidence that DKK1-CKAP4-associated signaling can interact with immune and tumor-microenvironmental processes in other gastrointestinal malignancies. A recent review by Despotidis et al. summarized links between DKK1-CKAP4 signaling and macrophage polarization, cytotoxic T-cell infiltration, antigen-presenting cells, and cytokine/chemokine signaling in preclinical GI cancer models [37], DOI: 10.1016/j.cpt.2026.09.002]. However, the present RNA-sequencing experiments were performed in EAC tumor-cell monocultures and therefore do not establish altered immune-cell function, tumor immunogenicity, or antitumor immunity. Accordingly, the observed changes should be interpreted as immune-related transcriptional alterations that warrant functional validation in models incorporating immune-cell interactions.

To validate the RNA sequencing data, selected differentially expressed genes were analyzed by qPCR. The significant upregulation of p21 and CX3CL1 following CKAP4 knockdown confirmed the transcriptomic findings. Increased p21 expression is consistent with the reduced proliferation observed in CKAP4-depleted cells, whereas elevated CX3CL1 further supports a role for CKAP4 in regulating immune-related signaling. In contrast, the expression changes of BCL2 and INHBA were not confirmed by qPCR, highlighting that not all transcriptomic findings could be reproduced by targeted gene expression analysis. Overall, these results strengthen the validity of the RNA sequencing data while emphasizing the importance of independent validation.

To explore whether CKAP4 depletion was accompanied by changes in DKK1 expression and downstream signaling, we analyzed DKK1 mRNA and protein levels in OE33 and OE19 cells following CKAP4-targeting siRNA treatment. CKAP4 depletion was associated with cell-line- and time-dependent increases in DKK1 mRNA expression, with a sustained increase in OE33 cells and a transient increase in OE19 cells at 48 h. In contrast, DKK1 protein levels remained unchanged in both cell lines, indicating that the observed transcriptional changes were not accompanied by corresponding alterations in DKK1 protein abundance. Although this discrepancy could reflect post-transcriptional regulation, the present data do not establish the underlying mechanism. Importantly, these findings do not provide direct evidence for a functional DKK1-CKAP4 signaling axis in EAC. Previous mechanistic evidence for DKK1-CKAP4-dependent signaling in esophageal cancer has been obtained primarily in ESCC, where DKK1/CKAP4-dependent AKT activation and proliferative effects have been demonstrated experimentally [37]. More recent literature also emphasizes that DKK1-CKAP4 signaling can be highly context-dependent across gastrointestinal malignancies, potentially reflecting differences in ligand availability, downstream effectors, and interacting signaling pathways [37]. Accordingly, the present EAC data should be interpreted as hypothesis-generating rather than as evidence establishing a functional DKK1-CKAP4 mechanism.

Regarding downstream signaling, CKAP4 knockdown did not consistently activate the PI3K/AKT pathway via DKK1 in EAC. Reduced AKT phosphorylation was observed only in OE19 cells, whereas OE33 cells showed no significant changes. In addition, no detectable changes were observed in the selected ß-catenin/GSK3ß readouts under the conditions tested, indicating that the canonical Wnt-pathway is not regulated by CKAP4 in these models.

Importantly, CKAP4 depletion had distinct effects on MAPK signaling, with a marked reduction in MAPK phosphorylation observed in OE33 cells. This suggests that CKAP4 may promote proliferation and survival in a context-dependent manner, potentially through MAPK signaling rather than the DKK1-PI3K/AKT axis.

The differential effects of CKAP4 depletion on AKT and MAPK signaling further support the presence of cell-line-specific signaling responses in EAC. CKAP4-targeting siRNA treatment was associated with reduced AKT phosphorylation predominantly in OE19 cells, whereas MAPK phosphorylation was mainly reduced in OE33 cells. These findings are consistent with previous reports indicating a greater dependence of OE33 cells on ERK/MAPK signaling for proliferation and invasion [38], PMID: 21143918; PMCID: PMC3009708], while OE19 cells appear to rely more strongly on PI3K/AKT-associated pathways [39], PMID: 29416661; PMCID: PMC5787518]. In line with this interpretation, a recent review by Despotidis et al. emphasized that DKK1-CKAP4-associated signaling can vary according to cellular context and tumor type, potentially reflecting differences in ligand availability, downstream effectors, and pathway crosstalk [37]. The same review also highlighted the potential for compensatory signaling through MAPK and PI3K-AKT/mTOR pathways following perturbation of the DKK1-CKAP4 axis [37]. Consistent with this heterogeneity, baseline AKT and p-AKT expression also differed among the esophageal carcinoma cell lines examined, including OE33, FLO-1, SK-GT-4, and OE19. Together, these observations support the biological plausibility of context-dependent CKAP4-associated signaling in EAC; however, they do not establish that the differential AKT and MAPK responses observed here are mediated by a functional DKK1-CKAP4 axis.

Future studies investigating the specific mechanisms controlling these responses and clarifying the role of different signaling pathways or post-translational modifications would be useful. A more detailed analysis of these differences could improve our understanding of CKAP4′s function in different cell types.

To further explore CKAP4 expression in external datasets, we analyzed publicly available patient cohorts and considered previously published clinical tissue data. In the GEO dataset, CKAP4 expression was lower in tumor tissue than in normal controls, whereas no difference was observed in the TCGA-ESCA cohort. These inconsistent findings indicate that CKAP4 expression is not uniformly increased across independent EAC datasets and may reflect differences in cohort composition, sample size, tumor heterogeneity, or technical factors related to data generation and normalization. Previously published immunohistochemical studies in ESCC reported low DKK1 and CKAP4 expression in adjacent non-neoplastic squamous mucosa and higher expression in tumor tissue, with co-expression associated with adverse clinical outcome [17]. However, these observations were obtained in ESCC rather than EAC and therefore cannot be used to validate the clinical or prognostic relevance of CKAP4 in EAC. Taken together, the available external data should be regarded as exploratory and do not establish CKAP4 as a clinically relevant biomarker in EAC.

The present study provides consistent in vitro evidence that CKAP4-targeting siRNA treatment is associated with reduced viability and proliferative capacity, altered expression of adhesion-related surface molecules, and cell-line-specific changes in intracellular signaling in EAC cells. These findings support further investigation of CKAP4 biology in EAC. At the same time, the use of established cell lines limits extrapolation to tumor behavior in vivo and to patients, and migration or metastatic behavior were not directly assessed. In addition, although the use of a heterogeneous siRNA pool reduces dependence on a single RNAi sequence, an independent non-overlapping CKAP4-targeting reagent or rescue experiment was not performed. Definitive attribution of all observed phenotypes specifically to CKAP4 therefore requires additional orthogonal validation. Future studies should include independent genetic perturbation or rescue approaches, together with clinically relevant in vivo or patient-derived models, to determine the functional and therapeutic significance of CKAP4 in EAC. Mechanistic studies will also be required to clarify whether the observed AKT and MAPK responses are mediated through DKK1-dependent CKAP4 signaling. In ESCC, Shinno et al. [17], provided stronger mechanistic evidence for a functional DKK1-CKAP4 axis using complementary knockdown, re-expression, and ligand-binding approaches; however, these findings were obtained in squamous cell carcinoma and cannot be directly extrapolated to EAC.

The immune-related transcriptional changes observed following CKAP4-targeting siRNA treatment provide an additional aspect of CKAP4-associated biology in EAC cells. RNA sequencing identified alterations in pathways related to inflammatory signaling, interferon responses, chemokine signaling, and antigen processing and presentation. Emerging evidence from other gastrointestinal tumor models suggests that DKK1-CKAP4-associated signaling can influence the tumor immune microenvironment, including macrophage polarization, antigen-presenting cell abundance, cytotoxic T-cell infiltration, and cytokine/chemokine signaling [37]. However, the present experiments were performed in EAC tumor-cell monocultures and did not include immune-cell co-culture or other functional immune assays. Therefore, the observed transcriptional changes cannot be interpreted as evidence of altered immune-cell behavior, tumor immunogenicity, or antitumor immunity. Functional validation in models incorporating tumor-immune cell interactions will be required to determine the biological significance of these immune-related transcriptional changes.

The present findings provide a rationale for evaluating CKAP4-associated phenotypes in more physiologically relevant EAC models. Future studies using xenograft, patient-derived xenograft, organoid, or other patient-derived systems will be important to determine whether the in vitro effects observed following CKAP4 depletion extend to tumor growth and signaling in vivo. Previous studies in ESCC, lung cancer, and pancreatic cancer have reported reduced tumor growth following CKAP4 depletion or antibody-mediated CKAP4 inhibition [13,15,17], providing broader preclinical support for further investigation of CKAP4-directed approaches. However, these findings were obtained in different tumor entities and do not establish therapeutic efficacy or safety of CKAP4 targeting in EAC. Similarly, although circulating CKAP4 has been investigated as a potential biomarker in ESCC [40], DOI: 10.2147/OTT.S183790], these data cannot be extrapolated to EAC. The present study did not assess CKAP4 expression in independent EAC tissue cohorts, serum samples, or patient outcome datasets and therefore cannot determine its diagnostic or prognostic value in this disease. Future studies should address these questions in independent, clinically annotated EAC cohorts and evaluate whether CKAP4 expression is associated with tumor characteristics, circulating protein levels, treatment response, or patient outcome.

## Conclusion

CKAP4 is detectable at the cell surface of EAC cell lines, and treatment with CKAP4-targeting siRNA was associated with reduced cell viability and proliferative capacity, an increased apoptotic fraction, cell-line-specific changes in AKT and MAPK signaling, altered surface expression of adhesion-related molecules, and immune-related transcriptional changes in vitro. The present data do not establish a uniform CKAP4-dependent signaling mechanism or a functional DKK1-CKAP4 axis in EAC. Moreover, the in vitro design and the absence of an independent non-overlapping CKAP4-targeting reagent, rescue experiments, and clinical validation preclude conclusions regarding therapeutic efficacy or biomarker utility. Further studies using orthogonal perturbation approaches and clinically relevant EAC models are required to define the mechanistic and translational significance of CKAP4 in this disease.

## Ethics approval and consent to participate

Not applicable.

## Consent for publication

Not applicable.

## Funding

This study was supported by the Barrett-Initiative e.V. and the Open Access Publishing Fund of Leipzig University, supported by the German Research Foundation within the program Open Access Publication Funding. This project has received funding from the European Union's Horizon Europe research and innovation program under the Marie Skłodowska-Curie grant agreement No 101119464.

## CRediT authorship contribution statement

**Beate Limberger:** Writing – review & editing, Writing – original draft, Visualization, Investigation, Formal analysis, Data curation. **Alexandra Ludwig:** Methodology, Data curation. **Philipp Walther:** Project administration, Data curation. **Chuang Yang:** Visualization, Investigation, Formal analysis. **Andreas Teufel:** Writing – review & editing, Resources. **Timo Itzel:** Writing – review & editing, Methodology, Investigation, Formal analysis. **Patrick S. Plum:** Writing – review & editing, Project administration, Conceptualization. **Orestis Lyros:** Writing – review & editing, Project administration, Conceptualization. **Stefan Niebisch:** Resources, Project administration. **Ines Gockel:** Writing – review & editing, Supervision, Project administration. **René Thieme:** Writing – review & editing, Writing – original draft, Supervision, Project administration, Funding acquisition, Conceptualization.

## Declaration of competing interest

The authors declare the following financial interests/personal relationships which may be considered as potential competing interests: Rene Thieme reports financial support was provided by University of Leipzig Faculty of Medicine. Rene Thieme reports a relationship with Barrett-Initiative e.V. that includes: funding grants. Rene Thieme reports a relationship with European Union that includes: funding grants. Rene Thieme reports a relationship with Owkin Inc that includes: funding grants. If there are other authors, they declare that they have no known competing financial interests or personal relationships that could have appeared to influence the work reported in this paper.

## Data Availability

The datasets generated during this study are available from the corresponding author upon reasonable request.
